# Northeastern Dental Association

**Published:** 1899-01

**Authors:** 


### NORTHEASTERN DENTAL ASSOCIATION.

The Fourth Annual Meeting of the Northeastern Dental Association was held in Hartford, Conn., Wednesday, October 19, 1898.

The meeting was called to order at eleven o'clock by President D. B. Ingalls, Clinton, Mass.

The records were read by the secretary, Dr. E. O. Kinsman, and accepted by the society.

*Dr. L. C. Taylor* (Hartford, Conn.).—Mr. President and gentlemen, I have been requested by not less than three of our society advocates to present the same subject here that I presented before the State Society. I am well aware that you may consider it ancient history, but it is history which interests us all. It is the matter of interstate registration. There is a growing unrest in the dental profession everywhere, caused by dentists who desire to go from one State to another, being obliged to pass a new examination before they are allowed to practise. This has had the mistaken effect of making all our laws unconstitutional. Dentists believe their State law is unconstitutional because the United States will not tolerate it. I want to sustain the State laws, and for that reason I have consented to go over the same ground that I did before. This was brought to my notice by a friend in Chicago. Permit me to read his letters. [Letters read.] They advocate forming laws which will allow a person who is a lawful practitioner in one State to have the same right in any other State, and advise the State of Connecticut to take up the matter.

And bearing on that point, I wish to read a little extract from what Dr. Truman said a year ago last August, at Old Point Comfort:

"The final outcome of this legal tendency has been laws in every State, a National Association of Dental Examiners, and a general feeling of disquiet among all teachers of dentistry as a consequence. Whether this product of the nineteenth century is to result in the good anticipated by those who worked for it remains to be seen; but it is hoped this Association will see the way clear to formulate something that will tend to quiet antagonism engendered by the unwise multiplication of statutes. The subject is a large one, and has frequently claimed the attention of my predecessors in office. As these laws at present stand, they are a dangerous obstacle in the path of professional progress, and promise to be the one blot upon the otherwise fair fame of the century in dental work. This applies not specially to individuals, for it is recognized that it is mainly the outgrowth of a mistaken sentiment that force, through law, will accomplish everything. The aim should be for unity of effort with the least friction, State with State, accompanied with a positive recognition that the decree of one State should in this matter be a law for every citizen of the United States. The Constitution of the United States expressly declares, Article IV., Section 1, 'Full faith and credit shall be given in each State to the public acts, records, and judicial proceedings of every other State, and the Congress may by general laws prescribe the manner in which such acts, records, and proceedings shall be proved, and the effect thereof.

"'Section 2. The citizens of each State shall be entitled to all the privileges and immunities of citizens of the several States.'

"These quotations are apparently clear, and unmistakably point to two facts,—that full faith and credit shall be given in each State to all the acts of every other State. . . . It is therefore clear that a law passed depriving a citizen who has been declared legally entitled to practise in any State from the privilege of registration in another State is unconstitutional, . . . and it is difficult to understand how the Supreme Court of the United States could decide otherwise if a case should be carried before it for adjudication."

These are the words of the president, and the National Dental Association at the first day's session offered a resolution stating that if a person in good standing presents a certificate from any State, he shall be allowed to practise in any other State without an additional examination. This was adopted. I do not propose to make long remarks, but I hope this will be brought forward after lunch, and that it will bring up quite a discussion when brought in legitimate form. I hope to have a committee appointed to put it in good shape. My thought is contained in these words: *Resolved*, That it is the opinion of this society that the different States shall make an interstate law, which shall conform to the Constitution of the United States by recognizing the judicial acts of all the other States, and admitting dentists to practise when the same right has been obtained in any one of the United States.

Dr. Charles McManus was here called upon to read his paper on "The Need of a Dental History."

### THE NEED OF A DENTAL HISTORY.

BY CHARLES M'MANUS, D.D.S.

A very few of you may remember that at the last meeting I called attention to a resolution adopted by the National Dental Association, "That a committee be appointed to report a measure looking to the preparation of a full history of the dental profession," and that on the strength of being chairman of that committee I read a paper entitled "One of the Present Needs of the Profession, a History."

I had hoped at that time to get an expression of opinion on this subject; but the paper, being presented with others after the evening banquet, was not discussed.

Well, a year has gone by, and the dental profession has been hard at work making all sorts of history; but the committee cannot report very much progress as yet in the matter of recording it. It is the intention, however, to make a full report on this subject at the meeting of the National Dental Association at Niagara Falls next August, and it is in the hope of awakening an interest in this really important matter that I bring it up now before this representative New England Society.

The early history of dentistry is to be found scattered here and there in the pages of old magazines and in a few privately printed pamphlets. It has that peculiarly dental quality of being inaccessible.

It has been gathered together in the following small books: by Duval, 1808; Fitch, 1829; Snell, 1832; Harris, 1849; Dexter, 1876; Cegrand, 1892–93; Lennmaln, 1895; Jacobi, 1896.

The list sounds rather more imposing than it really is, for Duval's work is in French, Fitch and Snell are rare and old books, Harris deals chiefly with biography, Jacobi's is a German work, and Lennmaln's is but a short compilation (as far as history is concerned) from Dexter.

This leaves Dexter's "History of Dental and Oral Science," which was published under the auspices of the American Academy of Dental Science twenty-two years ago, and is out of print, and Cegrand's "Rise, Fall, and Revival of Dental Prosthesis." The latter work was the outcome of several lectures delivered before the classes of a dental college in Chicago in 1892, and a second edition was published in 1893. It is a volume of three hundred and sixteen pages, and contains a great deal of curious and valuable information, and reflects credit on the author, who very modestly says in a letter, "I make no claim at having done a great work, but I do know that I have gathered numerous references, etc., and so arranged the book that he who wishes to go deeper into the subject will have had the many sources announced to him by foot-notes."

I wonder how many of the younger dentists of the country have read Cegrand's or Dexter's history, or in fact any history of dentistry, or are aware of what a gloriously independent history dentistry has had, and to what remarkable men we owe the making of the profession in this country.

It would seem that a more thorough realization and just pride in our past history might perhaps have a good influence on the professional tone of our future history.

What we need is an authoritative work on the subject that will carry it up to the present time, the close of the century.

As the editor of the *Dental Cosmos* says, "The history of dentistry has not yet been written, but it ought to be. The time is propitious, and the longer it is delayed the more difficult it will be to secure a reliable result. No single author could probably accomplish the task; but if a properly organized and concerted effort were made, the result could no doubt be attained. Let us hope that a creditable history of dentistry, not only of America, but of the whole world, may soon be forthcoming."

We have the men who can write that history right in the ranks of the dental profession, notably Dr. William H. Trueman, of Philadelphia, for example. Many of the men have helped materially to make that history. But the work must be done, or at least begun, before the close of this century, because, unfortunately, these older men are not growing younger. This fact was brought painfully to mind by the death, some months ago, of Dr. R. Finley Hunt, of Washington, a member of the Committee on History, and a venerable gentleman who had taken the greatest interest in the subject and was the originator of the present movement.

The great question is, How many men out of the twenty-five thousand individuals who are earning their living by dentistry will take any practical interest in this matter?

I once heard an authority upon the subject talk about the "ninety per cent. of dentists who are not endeavoring to elevate the profession." Can we count upon the two thousand five hundred men who are engaged in this elevating act? If we could feel sure that half that number, or even one thousand members of the profession, would take enough practical interest to signify a willingness, at the proper time, to subscribe for such a volume, the rest of the work would be comparatively easy.

It is a sad, sad fact that nearly everything connected with dentistry costs money, and it is sometimes said at dental meetings that if our patients want fine dentistry they will have to pay well for it. The same rule we can now apply to ourselves. We should have a work that should be thoroughly satisfactory from a professional, theoretical, and practical stand-point. To what extent will the dentists of New England go down into their pockets, at the proper time, to pay their share towards making it a success?

The following resolution was offered by Dr. C. W. Strang, and passed:

*Resolved*, That the Northeastern Dental Association, fully realizing the necessity for a complete and authoritative history of the dental profession, and believing that the present time is most auspicious, desires to express its hearty appreciation of the efforts now being made by the National Dental Association looking towards the preparation of this much-needed work.

Dr. C. W. Strang then read his paper on "Amalgam and Oxyphosphates as Filling-Material."

(For Dr. Strang's paper, see page 24.)

Dr. Hubbard, of the New York Dental School, read a paper on "Artificial Dentures in their Relation to the Speaking or the Singing Voice."

(For Dr. Hubbard's paper, see Vol. XIX., page 774.)

DISCUSSION.

*Dr. Flanagan* (Springfield, Mass.).—I think the time is coming when dentists are going to be called upon to assist vocal teachers.

Orators cannot gain the education here which they can across the water. The reason is that there they understand tone-formation. The paper of Dr. Hubbard has more in it than would appear on first observation. He has spoken of the tone-formation. We have all been to an ordinary play and heard a man sing. We may have a pleasing memory to carry away with us and we may not, and the whole will depend upon the tone-formation,—in other words, upon that small opening from which the tone comes. The teachers of vocal music will always impress upon their students the necessity of a clear formation in the mouth. Dr. Hubbard made use of a throaty formation, and he might refer to the man with the nasal twang. These different formations are caused by passing the sound through a place not proper for it. Again the man with the high tenor voice might be referred to. That man has gained a knowledge of how to place his tone. His tones vibrate. I certainly think it would be a benefit to have Dr. Hubbard's paper printed, because he has taken a step beyond all others.

Dr. D. D. Smith, of Philadelphia, read a paper on "Prophylaxis in Dentistry."

(For Dr. Smith's paper, see page 16.)

Dr. Smith prefaced his paper by remarking that the paper to which you have just listened forms a very excellent introduction to what I shall present to you. I will take the liberty of asking a question in regard to Dr. Hubbard's paper, which I hope will be answered some time before the close of the session. This subject, I understand, in its application to dentistry is not very well understood, but there are certain things which are understood. One is that a proper medium ought to be selected and employed by dentists when they are asked to assist in anything which might relate to the formation of sound. I would like to know what materials might be used. Dr. Hubbard spoke of vulcanite being a very poor material, and of the good properties of steel, but he said there is a happy medium to be found which will produce the best results. I do not understand how this is to be obtained, and it would be a source of gratification and information if we could have this distinctly brought up. It may be very well to theorize. I thought, as I came here to-day, that we seldom see at a meeting like this so many old men, men whose hair is as white as mine. Now, although we are of that class, we can be instructed, and I would very much like to be informed in regard to the dental application of that paper to which I have alluded. The subject which I have brought to you will perhaps not interest you, because it is not novel. It is on preservation or the warding off of decay.

The paper was then open for discussion.

*Dr. C. Frank Bliven* (Worcester, Mass.).—This paper has interested me most deeply. I followed the doctor very closely, and I endorse most heartily all that he says in regard to cleanliness. I think that cleanliness comes next to godliness, and that if a man is godly he will be clean. So it is with the mouths of those people who have reached a state of understanding. I rejoice most heartily with the doctor that he has found a panacea that will prevent decay in all teeth. I wish I had found, in my efforts to keep the teeth clean, a perfect prophylactic. Those who know and have seen the mouths under my care will say the mouths are clean. But I do not think the doctor has struck the key-note to this thing. There are certain teeth that it seems to me do not need cleaning,—that is, I cannot find any accumulation. Those teeth break down very rapidly. There is a cause for this, and it is our duty to see if we can discover it. In the past dentists have dealt with effects, and not with causes. When the patients come to us we seek the cavities and fill them with artificial substances. Do they accomplish the result desired? Those people who conscientiously use the new methods, do they accomplish the desired result? I leave the answer with you, who advocate these methods. We have to look still deeper for the cause and for the cure. Some dentists tell their patients that they eat food which is too hard, and which breaks the teeth or the fillings. Others will tell their patients to use hard food, that inorganic material will preserve the teeth. From my point of view, the protection must come from within the tooth, within the man.

It is essential that we have within the system just the properties that will continue to rebuild the bone. Now, I think I am safe in saying that the bone is rebuilt, because, throughout nature, waste from use is continually going on, and there must be a supply in order to rebuild, regenerate, support, and sustain. Hence it is necessary that we should have food that will do this for us. I am not advertising any particular form of food. It has been said that because I advocate the use of shredded wheat biscuits I have some financial interest in the company. There is not one atom of truth in that statement.

It seems to me that the basis of this thing must first rest upon a contented mind, because, so long as the mind is agitated, the functions of the system cannot go on properly, and the organs cannot perform their natural work of rebuilding as fast as they are wasted in use. We know that exercise and the use of every organ in our bodies is necessary. If you tie your arm to your side, in two or three weeks you would find it impossible to raise it to your face. To my mind, along with exercise and cleanliness should come contentment.

*Dr. D. D. Smith* (Philadelphia).—A matter which I overlooked is recalled to my mind by the last gentleman. He said that these accumulations were not on some teeth. On some teeth these accumulations are imperceptible oftentimes, but they are positively there, and you have only to try this process of thoroughly polishing the teeth to see what a change it makes in the teeth where the eye fails to detect the accumulations. But do not let all things degenerate to brushing the teeth. I am talking about stimulating the teeth by irritating the surface. Do not let it degenerate by saying that I have instructed my patients to brush their teeth. In regard to feeding that the pulp may do its work and protect the teeth, feed the teeth if you can, but I cannot uphold this plan. I can say, stimulate the pulp by irritation on the surface, and it will build up the teeth. I said to you what I believe to be true,—what I believe every one of the older members believe,—that feeding for the purpose of producing good teeth is a delusion and a snare. Not that the individual should not have good food. Give him good beef, good bread. Certainly he must have good food and everything the system demands and the system will take up, without any special material, for the teeth as for the muscles. There is no necessity, and it seems folly to me to feed the system with the view of making it produce good teeth. It simply cannot be done.

*Dr. S. S. Stowell* (Pittsfield, Mass.).—While Dr. Smith may be right theoretically, practically his plan is impossible. It would require twenty times the number of dentists there are to keep the people's mouths as clean as the doctor suggested. I do believe that the teeth are helped by having proper food, and I have written several articles on local exercise and put it in practice. I know it to be of use. In regard to the child which the doctor mentions, how does he know she would not have had sound teeth if they had not been treated in the way which they were? Cleanliness is important, but it is not everything, and will not remove the difficulty,—the tendency to decay.

*Dr. Bliven.*—We find that the cat and dog get along very nicely without having their teeth cleaned, for the reason that instinct leads them to select naturally organized food to rebuild their systems. Let me tell you of a case which I have under my charge. It is the case of a man whose teeth I have taken care of for some time. He has paid me from twenty-five to thirty dollars a year to keep his teeth in repair. They were well cleaned by himself. He is a farmer's son, and lived upon good food,—beef, potatoes, and nutritious things. But, nevertheless, this young man's teeth broke down rapidly. He left the farm to go West. Before he left he came to see me. I did everything I could for him, as he said he did not know when he would see a dentist again, and wanted to be protected.

Meeting adjourned.

(To be continued.)

